# Prevalence of Growth Restriction at Birth for Newborns With Congenital Heart Defects: A Population-Based Prospective Cohort Study EPICARD

**DOI:** 10.3389/fped.2021.676994

**Published:** 2021-05-28

**Authors:** Ali Ghanchi, Makan Rahshenas, Damien Bonnet, Neil Derridj, Nathalie LeLong, Laurent J. Salomon, Francois Goffinet, Babak Khoshnood

**Affiliations:** ^1^Université de Paris, CRESS, INSERM, INRA, Paris, France; ^2^Service d'Obstétrique - Maternité, Chirurgie Médecine et Imagerie Fœtales. APHP. Hôpital Necker Enfants Malades, Paris, France; ^3^Department of Pediatric Cardiology, M3C-Necker. APHP. Hôpital Necker-Enfants Malades, Paris, France; ^4^University of Paris, Paris, France; ^5^Port-Royal Maternity Unit, Cochin Hospital, APHP, Paris, France

**Keywords:** small for gestational age, congenital heart defects, population-based cohort, prevalence, ordinal logistic regression

## Abstract

**Background and Objectives:** Congenital heart defects (CHD) and growth restriction at birth are two major causes of childhood and adult morbidity and mortality. The aim of this study was to assess the overall risk of growth restriction at birth, as measured by its imperfect proxy small (< 10th percentile) for gestational age (SGA), for newborns with CHD.

**Methods:** Using data from a population-based cohort of children born with CHD, we assessed the risk of growth restriction at birth using SGA and severe SGA (3rd percentile). To compare the odds of SGA and severe SGA across five specific major CHD, we used ordinal logistic regression using isolated, minor (non-operated) ventricular septal defect (VSD) as the control group.

**Results:** The overall proportion of SGA for “isolated” CHD (i.e., those not associated with other anomalies) was 13% (95% CI, 12–15%), which is 30% higher than what would be expected in the general population (i.e., 10%). The risk of severe SGA was 5% (95% CI, 4–6%) as compared with the expected 3% in the general population. There were substantial differences in the risk of overall SGA and more so severe SGA across the different CHD. The highest risk of SGA occurred for Tetralogy of Fallot (adjusted OR 2.7, 95% CI, 1.3–5.8) and operated VSD (adjusted OR 2.1, 95% CI, 1.1–3.8) as compared with the control group of minor (non-operated) VSD.

**Conclusion:** The overall risks of both SGA and severe SGA were higher in isolated CHD than what would be expected in the general population with substantial differences across the subtypes of CHD. These results may provide a clue for understanding the underlying mechanisms of the relation between alterations in fetal circulation associated with different types of CHD and their effects on fetal growth.

## Introduction

Congenital heart defects (CHD) are the most frequent group of congenital anomalies with a prevalence of about 1% of all births (1, 2). Newborns with CHD are at a higher risk of growth restriction at birth (3–5). The latter may be an independent risk factor for adverse outcomes in newborns with CHD (5).

By far most of the previous studies that investigated the relation between CHD and growth restriction were hospital-based and population-based studies remain rare (3). Some of the literature has the shortcoming of including CHD associated with chromosomal or other anomalies without separate analyses of “isolated” CHD (not associated with chromosomal or other anomalies). Hence, the effects associated with the CHD *per se* are not always clear. Moreover, the specific effects of different types of CHD on the risk of growth restriction has not been adequately studied. Such an analysis may provide clues about the possible underlying mechanisms of the associations between CHD and growth restriction.

We used data from a population-based, prospective cohort study of more than 2,000 newborns with CHD to: (i) Assess the overall risk of growth restriction at birth for newborns with isolated CHD and to: (ii) Compare the risk and severity of growth restriction for five major types of CHD.

## Materials and Methods

### Data Source

The EPICARD study was a population based prospective cohort of children born with CHD in the Greater Paris area (Paris and its surrounding suburbs) of France carried out between 2005 and 2008.

All cases (live births, terminations of pregnancy for fetal anomaly, TOPFA and fetal deaths) diagnosed prenatally or up to 1 year of age were eligible for inclusion. Diagnoses of CHD and associated comorbidities (i.e., genetic, extra cardiac anomalies and/or syndromes) were confirmed by specialized pediatric cardiologists. Detailed description of the EPICARD cohort has been provided elsewhere (6).

**Figure 1:**
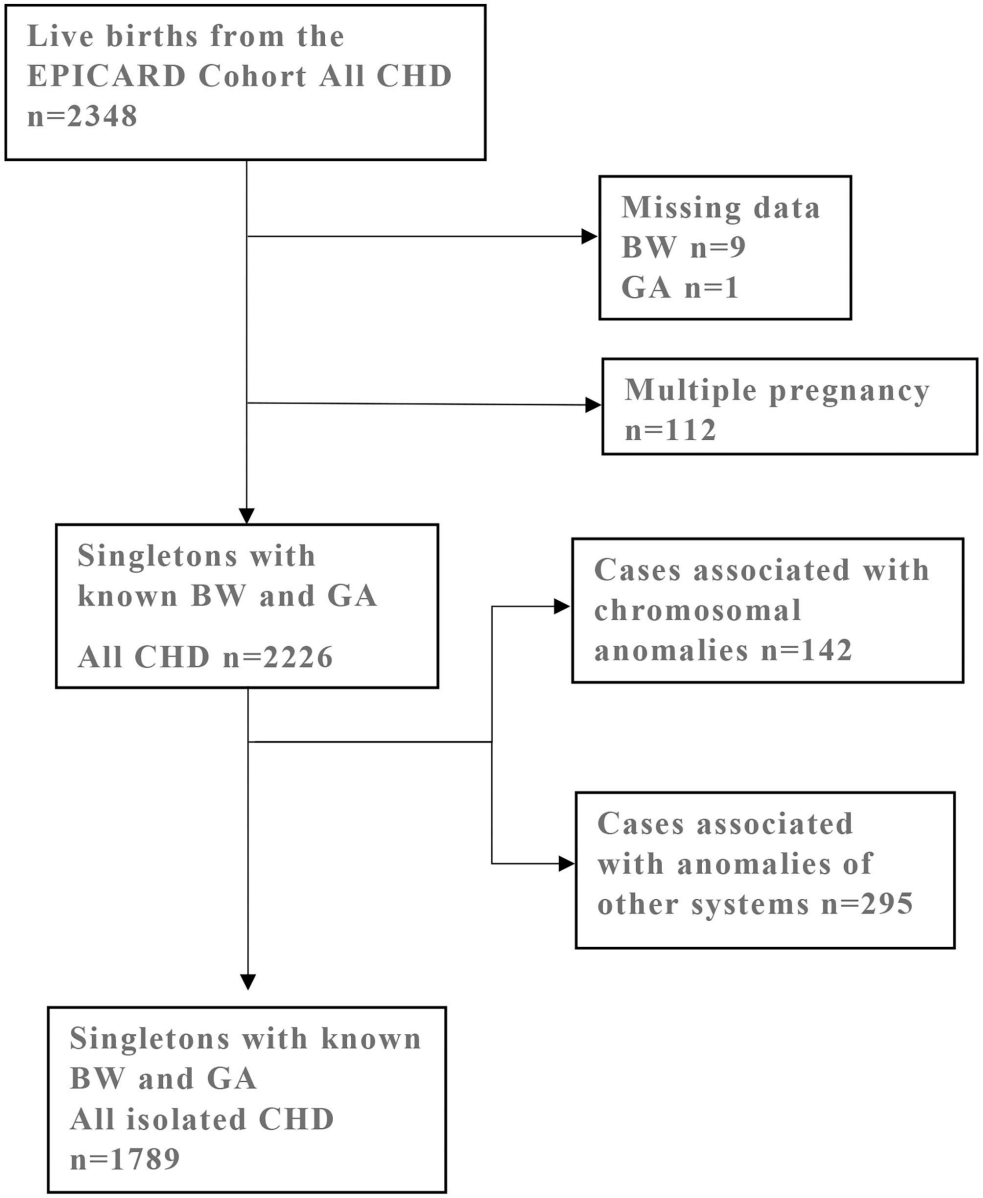
Selection of study population from the EPICARD Cohort of live birth born with all CHD. CHD, congenital heart defects; BW, birth weight; GA, gestational age.

From the EPICARD cohort of all live births (*N* = 2,348), we excluded 112 multiple pregnancies and ten subjects with missing data on birthweight and/or gestational age. Newborns with chromosomal anomalies (*n* = 142) or anomalies of other systems and/or genetic syndromes (*n* = 295) were also excluded from the study population. Our final study population comprised 1,789 singleton newborns with isolated CHD and known birthweight and gestational age (Figure 1).

### Outcome and Predictor Variables

The outcome variable, Small for Gestational Age (SGA) was defined as sex- and gestational age-specific birthweight <10th percentile based on the EPOPé population-based growth curves (7). We defined severe SGA using the cut-off birthweight <3rd percentile and Intermediate SGA as birthweights between the 3rd < 10th percentiles. Not -SGA was defined based on birthweight ≥ 10th percentile (8).

The main predictor variable of interest was type of CHD. We also took into account a set of potentially confounding variables including maternal diabetes, hypertension, smoking, maternal age, geographic origin, parity, prenatal diagnosis, infertility treatments, sex and preterm (< 37 weeks) delivery.

### Statistical Analysis

As the outcome variable comprised ordered outcomes (severe SGA/intermediate SGA/Not SGA) we used ordinal logistic regression for the statistical analysis. The proportional odds assumption for the ordinal logit models was tested and the models were found to be consistent with a proportional odds model.

The proportional odds model considers the cumulative probability of an individual event and all other events that are ordered before it (Box 1) (9, 10). Whereas, binary logistic regression uses the logit (log odds) function, ordinal logistic regression uses the logit transformation of the cumulative odds. In the proportional odds logit model, the slopes that correspond to the model coefficients are parallel to one another and the odds for each cut-off category differ only with regards to the intercept (Box 1). The χ^2^-test for the proportional odds assumption suggested that this was a reasonable assumption in the case of our models.

Box 1 **Ordinal logistic regression (9, 10).**

$Cumulative\;Odds\;\left(Y\leq j\right)=\frac{P\left(Y\leq j\right)}{1-P\left(Y\leq j\right)}\;$ *, j* = *1,…,k*

and in *logit* form :

$logit\left(Y\leq j\right)=\ln\left(\frac{P\left(Y\leq j\right)}{1-P\left(Y\leq j\right)}\right)$ *, j* = *1,…,k*

$logit\left(Y\leq j\right)=\alpha_{j}+X^{\prime }\beta$

Where:

*P*(*Y* ≤ *j*) = *P*_1_ + *P*_2_ + … + *P*_*j*_ is the cumulative probability of the event

α_*j*_: intercept parameters

β = (β_1_, β_2_, …, β_3_): a vector of unknown regression coefficients.

The statistical significance level was set at α = 0.05 and all analyses were done using Stata v15.1 software (StataCorp LP, College Station, TX, USA).

Ethics approval was obtained from the CNIL (Commission nationale de l'informatique et des libertés) (6).

## Results

Table 1 shows characteristics of the study population. Overall, 1,789 newborns with isolated CHD (not associated with chromosomal or other anomalies) were included in the study population. Of those, 47% were boys and 11% born preterm. Approximately 3% of women reported smoking during pregnancy, 1% had diabetes and 1% reported illicit drug use. Maternal age was 35 years or older for one quarter of women. One half of women were of French origin and 19% of North African origin. Approximately 7% of the study population were born after infertility treatments and 17% had a prenatal diagnosis of the CHD.

**Table 1:** Characteristics of the study population: EPICARD Study.

	***N***	**%**	**95% CI**
Sex			
Male	847	47	45–50
Preterm birth (< 37 weeks)			
Yes	192	11	9–12
Smoking during pregnancy			
Yes	50	3	2–4
Maternal diabetes			
Yes	25	1	1–2
Maternal illicit drug use			
Yes	10	1	0–1
Maternal age			
< 29	675	38	36–40
30-34	650	37	34–39
35-39	343	19	18–21
> 40	111	6	5–7
Parity			
0	638	36	34–38
1	545	31	29–33
> 2	595	33	31–36
Maternal geographic origin			
France	907	51	49–53
North Africa	330	19	17–20
Sub Saharan Africa	217	12	11–14
Other	329	18	17–20
Maternal high blood pressure			
Yes	22	1	1–2
Prenatal diagnosis of CHD			
Yes	313	17	16–19
Assisted reproductive technologies			
Yes	124	7	6–8
Small for gestational age			
Normal	1,554	87	85–88
< 10th percentile	235	13	12–15
3rd−10th percentile	142	8	7–9
< 3rd percentile	93	5	4–6
Birth weight (gr)	*Mean*	*SD*	
	3,175	618.71	3,147–3,204
Total number of patients	1,789		

Table 2 shows the proportions of SGA and severe SGA for isolated CHD and isolated specific CHD. The prevalence of SGA for isolated CHD was 13% (95%CI 12–15%) and 5% (95%CI 4–6%) for severe SGA. For specific CHD, SGA, ranged from 10% (95% CI 9–12%) for minor non-operated VSD to 26% (95% CI 16–40%) for Tetralogy of Fallot (ToF). Severe SGA proportions for specific CHD ranged from 4% (95% CI 3–5%) for non-operated VSD to 17% (95% CI 9–31%) for the ToF.

**Table 2:** Proportions of SGA (<10th percentile), intermediate SGA (≥ 3rd percentile <10th percentile), and severe SGA (<3rd percentile) for all isolated CHD, major isolated CHD, and isolated specific CHD.

		**SGA**			**Intermediate SGA**			**Severe SGA**		
	**Total**	***N***	**%**	**95% CI**	***N***	**%**	**95% CI**	***N***	**%**	**95% CI**
All isolated CHD	1,789	235	13	12–15	142	8	7–9	93	5	4–6
All isolated major CHD	493	78	16	13–19	51	10	8–13	27	6	4–8
Specific isolated CHD										
ToF	53	14	26	16–40	6	11	5–24	8	15	8–28
TGA	78	9	12	6–21	8	10	5–19	1	1	0–9
CoA	71	12	17	10–28	6	9	4–18	6	9	4–18
FUH	36	7	19	9–36	3	8	3–24	4	11	4–27
Operated VSD	128	27	21	15–29	16	13	8–20	11	9	5–15
Non-Operated VSD	1,063	113	11	9–13	69	6	5–8	44	4	3–6

*CHD, congenital heart defects; CoA, coarctation of the aorta; FUH, functionally univentricular heart; SGA, small for gestational age; TGA, transposition of the great arteries; ToF, Tetralogy of Fallot; VSD, ventricular septal defects*.

Table 3 shows the results of the ordinal logistic regression analysis for the five different types of CHD. There were substantial differences in the odds of both intermediate SGA and severe SGA across the five specific CHD.

**Table 3:** Odds ratios of SGA (severe and intermediate vs. normal) for different types of isolated CHD by ordinal logistic regression.

	**Crude odds ratio**	**95% CI**	Adjusted odds ratio^**^	**95% CI**
Minor ventricular septal defect (VSD)	Reference		Reference	
Operated ventricular septal defect (VSD)	2.8	1.6–4.8	2.0	1.1–3.8
Univentricular heart (UVH)	2.2	0.9–5.1	2.0	0.7–5.5
Tetralogy of Fallot (ToF)	3.3	1.7–6.2	2.7	1.3–5.8
Transposition of great arteries (TGA)	1.1	0.5–2.2	1.1	0.5–2.5
Coarctation of the aorta (CoA)	1.8	0.9–3.4	1.4	0.6–3.0

** *Adjusted on diabetes, maternal high blood pressure, maternal smoking during pregnancy, maternal geographic origin, parity, prenatal diagnosis, assisted reproductive therapy, gender and prematurity*.

In particular, the odds of overall and severe SGA were substantially higher for operated VSD and for ToF as compared with minor non-operated VSD; the adjusted odds ratios from the ordinal logit model were 2.1 (95% CI, 1.1–3.8) and 2.7 (95% CI, 1.3–5.8) for operated VSD and ToF, respectively.

## Discussion

Using population-based data from a large prospective cohort of children born with isolated CHD, we found that the overall prevalence of SGA was 13% and that of severe SGA 5%, both of which are higher than the expected proportions in the general population, 10 and 3%, respectively, based on the EPOPé population-based growth curves in France (7, 11).

We also found important differences in the probability of SGA and of severe SGA across the different types of CHD. In particular, VSD, which required surgery and Tetralogy of Fallot were associated with two- to three-folds higher odds of both intermediate and severe SGA, whereas minor VSD that did not require surgery was not associated with any significant increase in the risk of SGA as compared with the expected proportions in the general population. Whereas, newborns with SGA as a whole may include those who are constitutionally small, our findings were similar when we looked at severe SGA (birthweight <3rd percentile) and the latter is considered, by definition, to represent growth restriction at birth (12).

Our findings on the overall proportion of SGA for isolated CHD are comparable with a previous systematic review (3). However, previous data summarized in this systematic review did not allow estimates for severe SGA or for comparison of proportions of SGA across different types of CHD. Variations in the proportions of SGA and severe SGA for different CHD as reported in our study may provide insights into the pathophysiological mechanisms that link CHD with growth restriction at birth.

Two potential mechanisms may explain the relation between CHD and growth restriction at birth in general and in the case of differences across various types of CHD in particular. These include altered fetal hemodynamics and placental anomalies.

Matthieson et al. studied placental weight z scores in a Danish cohort of 7,569 children with CHD. They found that ToF and major VSD had lower placental weight which was in turn correlated with reduced birth weight and head circumference z scores (13).

Jones et al. found increased placental leptin secretion in children born with hypoplastic left heart syndrome and SGA (14). They argued that placental insufficiency results in SGA through reduced angiogenesis, which in turn reduces the surface area for gaseous exchange.

There may also be common etiological factors that cause both CHD and placental anomalies. Nitric oxide synthase (NOS) deficiency may be the common etiological factor that results in both CHD and fetal growth restriction. Liu et al. found that NOS was important in fetal heart development with deficiencies resulting in CHD (15). Other studies have shown that endothelial NOS may play an important role in fetal growth (16, 17).

Another possible mechanism is that alterations in blood flow circulation, result in differential perfusion and/or oxygen supply in the fetal body, which may in turn cause growth restriction in certain types of CHD but not necessarily others.

Wallenstein et al. found that CHD are associated with growth restriction and based on previous works by Rizzo et al. and Lutin et al., they argued that decreased ventricular output results in SGA but this may only occur in CHD with altered ventricular function (4, 18, 19). Using cardiovascular magnetic resonance, Al Nafsi et al. found that superior vena cava blood flow varied in left sided CHD compared to controls without CHD (20). Story et al. also found differences in the proportion of SGA in specific CHD, notably 13% SGA in TGA, 17% SGA in CoA and 26% SGA in ToF (results similar to those in our study) (5). The authors hypothesized that growth restriction for ToF was due to decreased fetal blood flow and hence reduced oxygenation. This may only be true however, in case of fetal heart failure or when the arterial duct is absent or closed. They also reported that newborns with CoA have decreased overall birthweight and length but normal head circumference and a greater head volume to birthweight ratio. The latter may be due to decreased caudal blood flow (without a decrease in oxygen saturation.

Donfrio et al. used Doppler ultra sound to demonstrate that decreased blood oxygenation due to abnormal fetal hemodynamics results in enhanced cerebral blood perfusion (brain sparing effect) as an adaptive compensatory mechanism in single ventricle defects (hypolastic left and right syndromes) (21). However, enhanced cerebral perfusion occurs at the expense of fetal liver, renal, pancreatic and mesenteric circulation, which in turn results in decreased production of insulin growth factor, angiotensin and other endocrine hormones essential for fetal growth (22). These hormones may affect the fetal growth directly as is the case of insulin growth factor or indirectly via placental function (e.g., renin-angiotensin system), inducing an inflammatory response or through other biomolecular pathways (23, 24).

Alternatively, abnormal fetal hemodynamics may affect the placenta resulting in SGA either through elevated fetoplacental vascular resistance due to placental ischemia or by fetoplacental endothelial dysregulation which plays a key role in tempering inflammatory regulators and nutrient exchange (25). Nevertheless, in general, the underlying pathophysiological mechanisms of the associations (or lack thereof) between CHD and the risk of growth restriction are complex and most likely involve multifactorial causal pathways and compensatory mechanisms that are not completely understood. Moreover, in addition to the placental and fetal hemodynamic mechanisms, there are also genetic and epigenetics factors related to the risk of growth restriction with CHD (26–28).

Our study has certain limits. Our study was not designed and cannot disentangle the possible mechanisms that may explain our empirical findings. Indeed, investigation of the possible underlying mechanisms of the relation between CHD and fetal growth were beyond the scope or ambition of our study.

In addition, even if data were from a large, population-based of newborns with CHD, the number of cases for individual specific CHD was relatively small, which resulted in reduced precision in our estimates, particularly for the fortunately less common severe SGA outcomes. However, by using the ordinal logit model, which allowed looking at both severe and intermediate SGA outcomes in the same model, we were able to increase the statistical power of our study, including for severe SGA outcome (9, 10).

### Conclusion

Congenital heart defects are associated with a higher risk of growth restriction at birth, including a higher risk of severe growth restriction.

The risk of growth restriction was substantially higher for certain types of CHD, notably operated ventricular septal defects and the Tetralogy of Fallot. The underlying mechanisms of the relation between CHD and growth restriction at birth may be hypoxia and alternations in blood perfusion in the fetus. In addition, placenta is likely to play an important role in the causal links between CHD and fetal growth restriction. Future studies are needed to disentangle the underlying mechanisms, including genetic and epigenetic factors that may explain the higher risk of growth restriction for newborns with congenital heart defects.

## Data Availability Statement

The original contributions presented in the study are included in the article/supplementary material, further inquiries can be directed to the corresponding author/s.

## Ethics Statement

The studies involving human participants were reviewed and approved by CNIL (French National Committee of information and Liberty). Written informed consent to participate in this study was provided by the participants' legal guardian/next of kin.

## Epicard Study Group

Principal Investigators: François Goffinet, Babak Khoshnood.

Steering Committee: Damien Bonnet (Hôpital Necker Enfants Malades, AP-HP, Center de référence M3C, Université Paris Descartes, Paris), Johanna Calderon (INSERM U1153), Drina Candilis (Université Paris-Diderot, Paris), Anne-Lise Delezoide (Hôpital Robert Debré, AP-HP, Service de biologie du Développement, Université Paris-Diderot, Paris), Catherine De Vigan (INSERM 1153, Paris), François Goffinet (Groupe Hospitalier Cochin-Hôtel Dieu, AP-HP, Maternité Port-Royal et INSERM U953, Université Paris Descartes, Paris), Lucile Houyel (Hôpital Marie Lannelongue, Service de chirurgie des cardiopathies congénitales, Le Plessis-Robinson), Jean-Marie Jouannic (Hôpital Trousseau, AP-HP, Center pluridisciplinaire de diagnostic prénatal, UPMC, Paris), Babak Khoshnood (INSERM U1153, Paris), Nathalie Lelong (INSERM U1153, Paris), Suzel Magnier (Hôpital Robert Debré, AP-HP, Service de cardiologie, Paris), Jean-François Magny (Institut de Puériculture et de périnatologie, Service de néonatologie, Paris), Caroline Rambaud (Hôpital Raymond Poincarré, AP-HP, Service d'anatomie et cytologie pathologiques – Médecine légale, UVSQ, Garches), Dominique Salomon (INSERM U1153, Paris), Véronique Vodovar (INSERM U1153, Paris).

Project Coordination and Data Analysis Committee: François Goffinet, Babak Khoshnood, Nathalie Lelong, Anne-Claire Thieulin, Thibaut Andrieu, Véronique Vodovar.

Independent Data Monitoring Committee (URC Paris Center et CIC Cochin Necker Mère Enfant): Maggy Chausson, Anissa Brinis, Laure Faure, Maryline Delattre, Jean-Marc Treluyer (Groupe Hospitalier Cochin-Hôtel Dieu, AP-HP, Université Paris Descartes, Paris).

External Scientific Committee: Gérard Bréart, Dominique Cabrol, Alain Sérraf, Daniel Sidi, Marcel Voyer.

Participating Centers: The Greater Paris Area (Paris and its surrounding suburbs) public (AP-HP) and private maternity units, Departments of Pediatric Cardiology and Pediatric Cardiac Surgery, pediatric cardiologists in private practice, Neonatal Intensive Care Units, Pediatric Intensive Care Units, Emergency Transfer Services (SMUR), Departments of Pathology, Sudden Death Centers, Departments of Family and Infant Protection (DFPE).

## Author Contributions

BK conceived the study. AG conducted the statistical analyses and wrote the first draft of the manuscript. DB, FG, and BK contributed to the conceptualization of ideas and made suggestions about the required analyses. All authors contributed to the interpretation of findings and revisions of the article.

## Conflict of Interest

The authors declare that the research was conducted in the absence of any commercial or financial relationships that could be construed as a potential conflict of interest.

## Abbreviations

CHD: Congenital Heart defects
VSD: Ventricular Septal Defects
CoA: Coarctation of the Aorta
ToF: Tetralogy of Fallot
TGA: Transposition of the Great Arteries
FUH: Functionally Univentricular Heart
SGA: Small for Gestational Age
TOPFA: Termination of Pregnancy for Fetal anomaly.
